# Effects of olfactory and/or gustatory stimuli on feeding of preterm infants: A systematic review and meta-analysis

**DOI:** 10.1371/journal.pone.0301186

**Published:** 2024-05-07

**Authors:** Deping Zhang, Qizhen Lu, Li Li, Xiaofeng Wang

**Affiliations:** 1 Department of Operating Theatre, The First Hospital of China Medical University, Shenyang, China; 2 Department of Operating Room, The First Hospital of China Medical University, Shenyang, China; 3 Central Sterile Supply Department, The First Hospital of China Medical University, Shenyang, China; 4 Mammography, Liaoning Cancer Hospital, Shenyang, China; Universiti Malaya Fakulti Perubatan: University of Malaya Faculty of Medicine, MALAYSIA

## Abstract

**Aim:**

To evaluate the effect of olfactory and/or gustatory stimulation interventions on feeding outcomes in preterm infants.

**Methods:**

We conducted systematic searches across various academic databases, including PubMed, Embase, Web of Science, the Cochrane Library, the Chinese Biomedical Literature Service System, China National Knowledge Infrastructure, the Wanfang Database, and the Wipu Database. These searches aimed to identify randomized controlled trials investigating the impact of olfactory and/or gustatory stimulation on preterm infants. The search period spanned from the inception of the databases until December 2022. Two independent evaluators autonomously reviewed the literature, extracted pertinent data, assessed the quality of the included studies, and conducted a meta-analysis using RevMan 5.3 software.

**Results:**

A total of 7 randomized controlled trials or quasi-experimental studies were included, with a total of 871 participants. Olfactory and gustatory stimulation demonstrated a reduction in the time to full enteral feeds in preterm infants when compared to usual care (MD = -1.60 days; 95% CI = -2.31, -0.89; p<0.0001). No substantial evidence was identified regarding the influence of olfactory and gustatory stimulation on the duration of gastric tube placement, length of hospitalization, incidence of necrotizing enterocolitis, or occurrence of spontaneous bowel perforation in preterm infants.

**Conclusions:**

Olfactory and gustatory stimulation show potential benefits for preterm infants. However, due to the low to very low level of certainty associated with the available data, our ability to assess the effects is limited. Further trials and studies are essential to enhance our understanding of the mechanisms and effectiveness of olfactory and gustatory stimulation therapies.

## 1. Background

Preterm infants (< 37 weeks) commonly struggle to synchronize sucking, swallowing, and breathing as a result of the immaturity of their neurological and digestive systems [1]. Hence, the majority of preterm newborns require parenteral nutrition or nasogastric or orogastric tube feeding to deliver enough nutrition in the interest of maximizing nutritional support [2]. However, preterm infants are still susceptible to complications even when receiving the aforementioned treatments, with prolonged intravenous nutrition increasing the risk of parenteral nutrition-associated liver disease, central cannula-associated bloodstream infections, and the beginning of cannula feeding increasing the risk of necrotizing enterocolitis and spontaneous intestinal perforation, which have a detrimental effect on infant survival [3, 4]. Thereby, research has turned to interventions to hasten the transition of premature newborns to suckle feeding.

The intense sensory inputs of smell and taste are crucial for starting the metabolic pathways that help with digestion and metabolic control [5]. The digestive process initiates before the food entering the stomach. This is due to the olfactory and gustatory senses triggering neurological responses that facilitate salivation, osmotic movements, and the secretion of crucial hormones and chemicals necessary for digestion [6]. These include glucagon, leptin, ghrelin, pancreatic polypeptide, and gastrin [7]. These reflexes are described as having a "cephalic phase response" [8]. Pavlov initially proposed this theory in his work on the digestive tract and its secretions, which focused on how sensory contact with food influences the cephalic digestive response [9]. As a result, adding olfactory and gustatory cues to preterm infants before they begin eating may boost this response. The olfactory and gustatory receptors of the infant begin to develop at 8 weeks into the pregnancy and finish maturing at 24 and 17 weeks, respectively [10]. Moreover, preterm infants’ behavioral reactions to odors have shown them to be olfactory sensitive [11]. The functions of smell and taste are, however, disregarded when preterm newborns get nutrients through tubes that skip the nose and mouth cavities [12]. Additionally, infants who are tube-fed do not experience typical eating habits from an early age (sucking, swallowing, and chewing), which may have an impact on the physiological development of those behaviors and cause delays in all-enteral feeding and suckling feeding, both of which can be harmful to the infant’s transitional recovery [2, 13, 14].

A recent Cochrane systematic review investigated the efficacy of exposing premature infants to milk odor or flavor, or both, delivered via a tube, and found no significant impact on the duration of achieving complete suckle feeding [12]. However, only two studies were included in this review in a quantitative synthesis, a small number of studies included, and low quality of evidence. As a result, a rather more complete and up-to-date meta-analysis was carried out to improve our understanding of the efficacy of such interventions.

## 2 Methods

### 2.1 Objectives

The goal of this systematic review and meta-analysis was to investigate the effect of olfactory and/or gustatory stimulation therapies on feeding outcomes in premature infants.

### 2.2 Design

The Preferred Reporting Items for Systematic Reviews and Meta-Analyses (PRISMA) criteria [15] and the Cochrane Handbook for Systematic Reviews of Interventions [16] were used to perform this systematic review and meta-analysis. You may find the protocol on PROSPERO (CRD42023385687).

### 2.3 Search methods

#### 2.3.1 Inclusion and exclusion criteria

According to the PICOS model, the inclusion criteria were determined as follows: P: preterm infants (born before 37 weeks of gestation); I: exposing premature infants to the sensory attributes of breastfeeding, including its taste and aroma, as well as those of commercial infant formula. C: standard of care or exposure to odorless, tasteless distilled water; O: time to full enteral feeding (at least 24 consecutive hours of nasogastric nutrition at a rate of 120 mL per kilogram of body weight) and duration of parenteral nutrition (i.e., the time to remove the parenteral nutrition tube); S: study design containing a randomized controlled trial or quasi-experimental studies. Moreover, we did not include any studies that fulfilled even one of the following requirements: (a) Duplicate publications; (b) unavailable full texts; (c) incomplete data reported in the study, or data that could not be extracted from the database.

#### 2.3.2 Search strategy

We conducted a systematic search of PubMed, Embase, the Web of Science, the Cochrane Library, the Chinese Biomedical Literature Service System, China National Knowledge Infrastructure, the Wanfang Database, and the Wipu Database to identify studies published until December 2022. The search was not restricted by geographical or language constraints. We used both free-text keywords and Medical Subject Headings (MeSH) to organize our search algorithms. The keywords included smell, odor, taste, gustation, and milk; breast, human milk, or infant formula; and premature infant, preterm infant, or neonatal prematurity. For specific examples of our search strategies, please refer to S1 File. To identify additional relevant information, we manually reviewed the reference lists of previously published reviews and selected publications. Two authors independently validated the search findings by searching the selected databases.

### 2.4 Study selection and data extraction

Following the predefined inclusion and exclusion criteria, two researchers conducted an independent selection of the studies. They carefully evaluated the complete texts of potentially eligible papers after reviewing the titles and abstracts. The information collected from these studies included titles, year of publication, journal, first author, sample size, research methodology, participant baseline characteristics, intervention strategies, and findings. In cases where there were disagreements between the two reviewers, a third reviewer was involved to help resolve the differences and reach a consensus. This process ensured a comprehensive and rigorous evaluation of the selected studies.

### 2.5 Quality appraisal

The literature’s quality underwent assessment using the risk of bias assessment tool version 2.0 (RoB 2.0) recommended by the Cochrane Systematic Evaluator’s Manual 6.1 [17]. Two researchers summarized the risk of bias assessment, evaluating potential biases in the included literature across the following domains: randomization process, deviation from the intended interventions, missing outcomes, measurement of the outcome, and selection of reported results. In instances of disagreement during the evaluation, resolution was achieved through discussion and negotiation.

### 2.6 Quality of evidence

Three reviewers (AB, AL, and GD) utilized the Grading of Recommendations Assessment, Development, and Evaluation (GRADE) working group methodology to assess the certainty of evidence across studies and assign ratings ranging from very low to high. The GRADE framework evaluates the certainty of evidence based on factors such as study design, risk of bias, inconsistency, indirectness, and imprecision. The certainty of evidence is categorized into levels ranging from very low to high, these criteria were assessed based on standardized scores that account for factors influencing evidence quality, either reducing or enhancing it [18, 19]. Refer to S2 File for particular assessment standards.

### 2.7 Data synthesis

The statistical analysis was conducted using RevMan 5.3 software. For continuous variables, we utilized either the standardized mean difference (SMD) or the weighted mean difference (MD) analysis. Random or fixed effects models were chosen based on whether the intervention effects were likely to be the same (preferred fixed effects) or not (random effects) [20]. We categorize heterogeneity according to its magnitude into four degrees as follows: 0% to 40%: might not be important; 30% to 60% may represent moderate heterogeneity; 50% to 90% may represent substantial heterogeneity; 75% to 100% may represent considerable heterogeneity. Due to the limited number of studies included in the literature, we only conducted subgroup analyses on the time to full enteral feeds for outcome indicators based on variations in olfactory stimulation or taste stimulation. In addition, funnel plots were used to assess potential publication bias when the study group contained 10 or more included publications.

## 3 Results

### 3.1 Search outcomes

**Fig 1:**
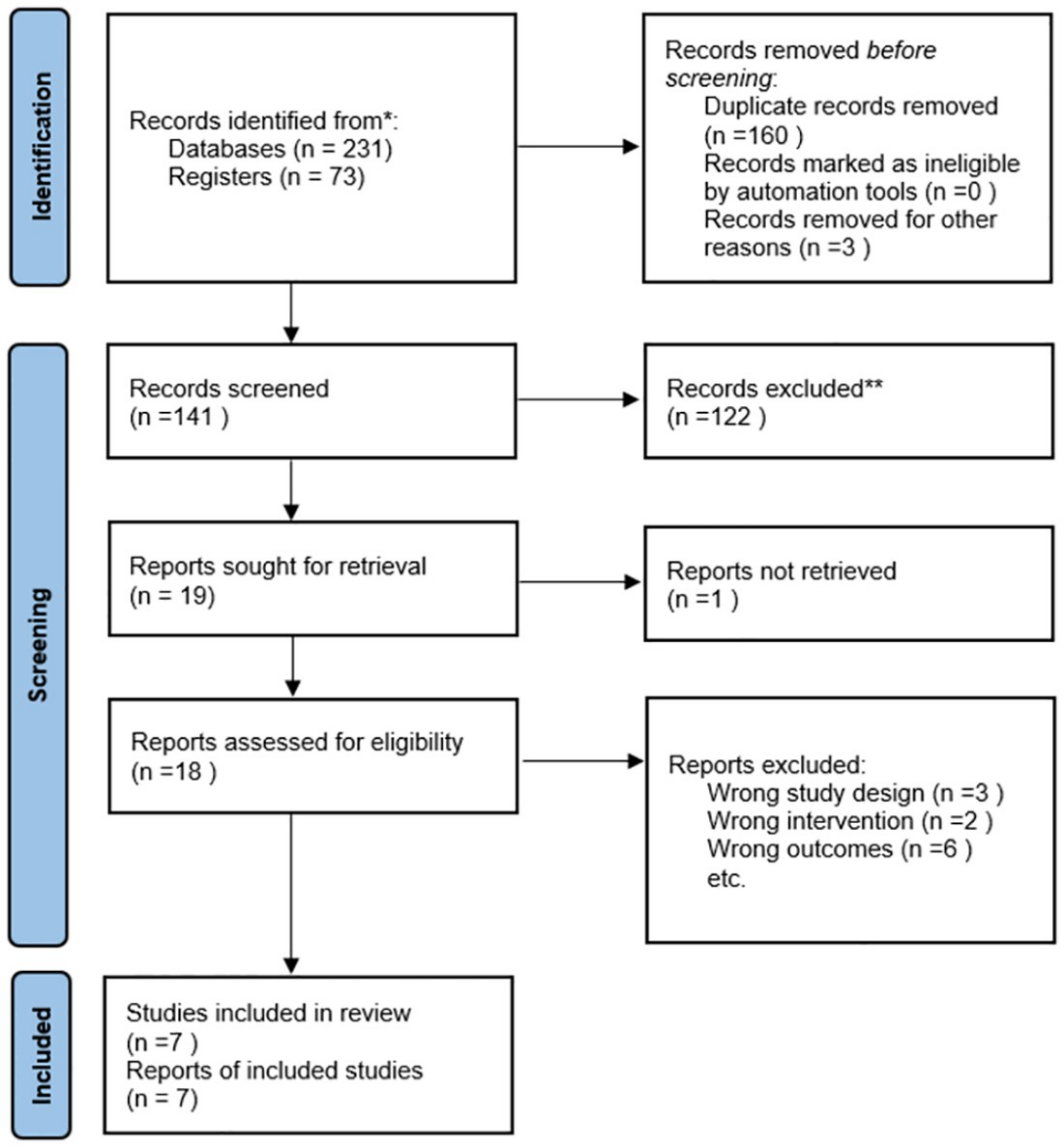
PRISMA flow diagram of literature search and selection process.

A total of 304 articles were initially collected in the search. Among these, 163 duplicate articles were removed using Endnote software. Upon reviewing the titles and abstracts, 122 articles were further excluded. Following a thorough examination of the full texts, an additional 12 articles were eliminated due to discrepancies in study design, intervention methods, and outcome indicators. Ultimately, the meta-analysis was conducted using a set of 7 publications. Fig 1 presents a flowchart illustrating the specific screening process according to the PRISMA guidelines.

### 3.2 Characteristics of the studies

A total of 871 premature infants were involved in the seven studies that were included [10, 21–26], with sample sizes ranging from 16 to 200. Among the included studies were one quasi-experimental research study and six two-arm randomized controlled trials. [10, 21–25]. Between 2011 and 2022, the research was published. Two of the studies were carried out in Australia, two in China, one in Turkey, one in Iran, and one in America. The mean gestational age of preterm infants in this systematic review ranged from 26.9 to 34.1 weeks. The features of the seven studies were summarized in Table 1. Two studies performed only olfactory stimulation of breast milk odor and did not perform gustatory stimulation [24, 26]. Preterm infants had access to non-nutritive sucking in four research interventions [10, 21, 23, 25]. (See Table 1).

**Table 1:** Summary table of included studies.

Author,year	Country	Study design	Participants(intervention/control)	Gender (female)	Intervention	Control	Frequency of stimulation	Birth weight (SD OR IQR)	Birth weight (g)	Outcome
Beker2017	Australia	RCT, two parallel groups	28/23	52%	smell and taste stimulation groups	Routine care group		26,95±1.45	939.5±215.5	Time to full enteral feeds, Duration of parenteral nutrition, Necrotising enterocolitis, Spontaneous intestinal perforation, Discharge weight, PMA at discharge
Beker2021	Australia	RCT, two parallel groups	196/200	48.50%	smell and taste stimulation groups	Routine care group		27.6±2.2	939±228	Time to full enteral feeds, Duration of parenteral nutrition, Necrotising enterocolitis, Spontaneous intestinal perforation, Discharge weight, PMA at discharge
Davidson2019	American	RCT, two parallel groups	17/16	52%	mother’s own milk (MOM) group	water (sham) stimulus	4–6 times per week, until either the infant reached full oral feeds or was transferred	30.5		Time to full enteral feeds, PMA at discharge
Dongmei2022	China	RCT, two parallel groups	57/57	45%	smell and taste stimulation groups	Routine care group	3 times/day until preterm infant can suckle independently	31.64±1.61	1670±375	Time to full enteral feeds, Duration of parenteral nutrition, Duration of gastric tube placement, Length of stay in hospital, Discharge weight
Leqiong2021	China	RCT, two parallel groups	89/76	52%	Smell and taste group	Routine care group	3 times/day until gastric tube is removed	34.08±1.42	1689.89±229.92	Time to full enteral feeds, Duration of parenteral nutrition, Duration of gastric tube placement, Length of stay in hospital, Necrotising enterocolitis, Spontaneous intestinal perforation
Yildiz2011	Turkey	quasi-experimental study	40/40	48%	mother milk odor	Routine care group	three feedings until the infant graduated to oral feeding	31.16±1.45	1536±339	Time to full enteral feeds, Length of stay in hospital, Discharge weight
Khodagholi2019	Iran	RCT, two parallel groups	16/16	56%	smell and taste stimulation groups	non-nutritive sucking (NNS) group	3 times/day for 10 days	29.2±1.31	1316±153.5	Length of stay in hospital, Discharge weight, PMA at discharge

### 3.3 Risk of bias

**Fig 2:**
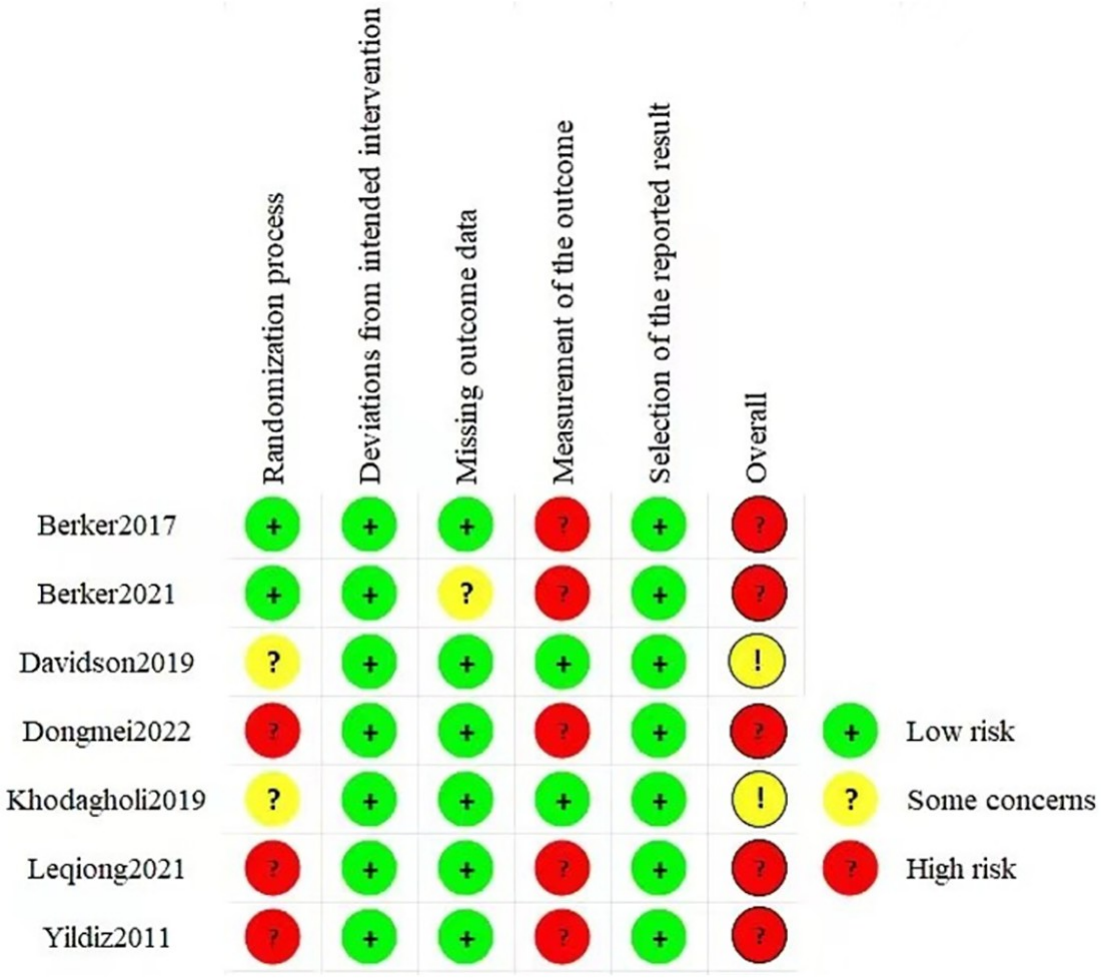
Assessment of risk of bias of the included studies.

In the context of randomization bias, two studies [10, 23] provided comprehensive descriptions of the randomization method and allocation concealment. All studies demonstrated a low risk of bias in terms of deviation from the intended interventions and selection of reported results. Except for the study conducted by Berker et al. [10], all other studies exhibited a low risk of bias concerning missing outcomes. According to the evaluation criteria, two studies [24, 25] raised some concerns regarding the overall risk of bias, while the remaining studies were deemed to have a high risk of bias. Fig 2 displays an overview of bias risk.

### 3.4 Results of the meta-analysis

#### 3.4.1 Time to full enteral feeds

**Fig 3:**
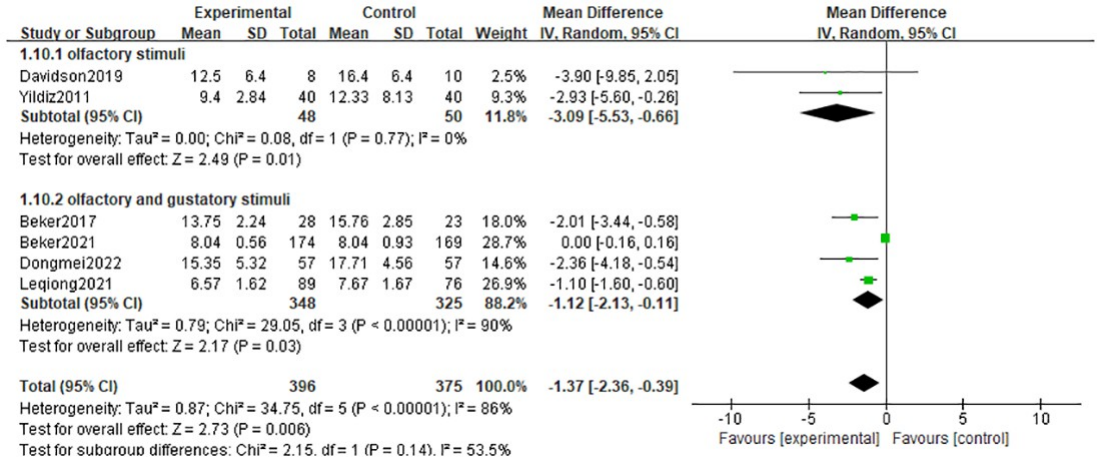
Forest plot for time to full enteral feeds.

The analysis incorporated six studies [10, 21–24, 26] that examined outcome indicators related to time to full enteral feeds. The results of the random-effects model showed that interventions that stimulated the olfactory and/or gustatory systems were significantly better than controls in shortening time to full enteral feeds (MD = -1.37 days; 95% CI = -2.36, -0.39; p = 0.006), and there was considerable heterogeneity (I^2^ = 86%, p < 0.00001). To explore heterogeneity, we performed subgroup analyses according to intervention modality, and olfactory stimulation alone shortened time to full enteral feeds compared with usual care (MD = -3.09 days; 95% CI = -5.53, -0.66; I^2^ = 0%, p = 0.01). Concurrent use of olfactory and gustatory stimuli similarly shortened time to full enteral feeds (MD = -1.12 days; 95% CI = -2.13, -0.11; I^2^ = 90%, p = 0.03). (Fig 3). Of these, there was still considerable heterogeneity in the olfactory and gustatory stimulation groups, which may be related to the lower gestational age of the preterm infants included in Beker et al [10, 23]. In addition, we performed a sensitivity analysis of this result using fixed effects, which showed that inconsistency in the results arose after excluding the study by Beker et al [23]. This may be related to the large sample size and high weighting percentage of this study.

#### 3.4.2 Duration of parenteral nutrition

**Fig 4:**
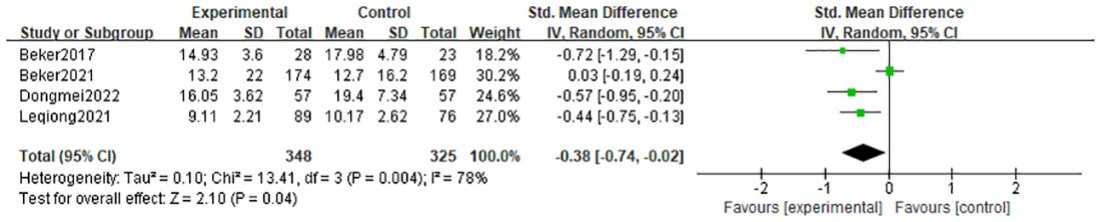
Forest plot for duration of parenteral nutrition.

In four studies [10, 21–23], the length of parenteral feeding was listed as an outcome. The random-effects model’s findings revealed that interventions that stimulated the olfactory and/or gustatory systems were significantly more effective than controls at reducing the length of parenteral nutrition (MD = -1.89 days; 95% CI = -3.40, -0.39; p = 0.01) and that there is substantial heterogeneity (I^2^ = 78%, p = 0.004) in Fig 4.

#### 3.4.3 Length of stay in hospital

**Fig 5:**
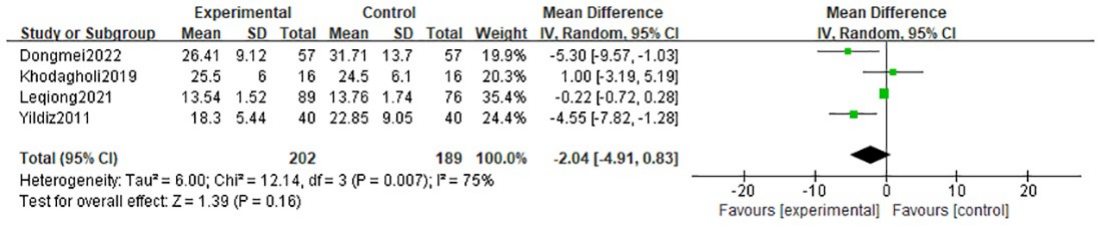
Forest plot for length of stay in hospital.

The length of stay in the hospital was used as an outcome indicator in four studies [21, 22, 25, 26], and the results of a random effects model indicated that there was no significant difference in length of stay between the olfactory and/or gustatory stimulation group and the control group (MD = -2.04 days; 95% CI = -4.91, -0.83; p = 0.16), and only substantial heterogeneity (I^2^ = 75%, p = 0.007) in Fig 5.

#### 3.4.4 Discharge weight

**Fig 6:**
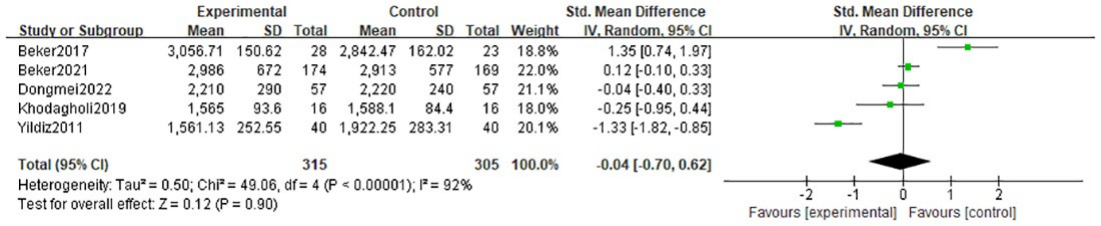
Forest plot for discharge weight.

Discharge weight was used as an outcome indicator in five studies [10, 21, 23, 25, 26], where considerable heterogeneity was found (I^2^ = 92%, p<0.00001). When olfactory and/or gustatory stimulation was compared to standard care, there was no statistically significant difference in the discharge weight between the two groups (SMD = 0.04g; 95% CI = -0.70, 0.62; p = 0.90). In Fig 6.

#### 3.4.5 Duration of gastric tube placement

**Fig 7:**
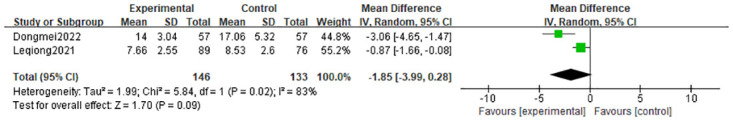
Forest plot for the duration of gastric tube placement.

Two studies [21, 22] reported on the length of gastric tube insertion as an outcome indicator, revealing considerable heterogeneity (I^2^ = 83%, p = 0.02). A random effects model analysis found no significant difference in the duration of gastric tube placement between the olfactory and/or gustatory stimulation groups and routine care. (MD = -1.85 days; 95% CI = -3.99, 0.28; p = 0.09). (In Fig 7).

#### 3.4.6 Necrotizing enterocolitis

**Fig 8:**
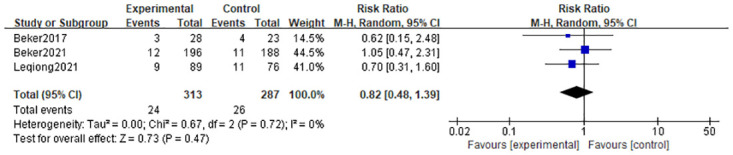
Forest plot for necrotising enterocolitis.

There was no difference in the relative risk of necrotizing enterocolitis in the olfactory and/or gustatory stimulation group compared to standard care (RR = 0.82; 95% CI = 0.48; 1.39; P = 0.47); and there was no heterogeneity (I^2^ = 0%, P = 0.72) in three studies [10, 22, 23] that reported on the incidence of necrotizing enterocolitis. (In Fig 8).

#### 3.4.7 Spontaneous intestinal perforation

**Fig 9:**
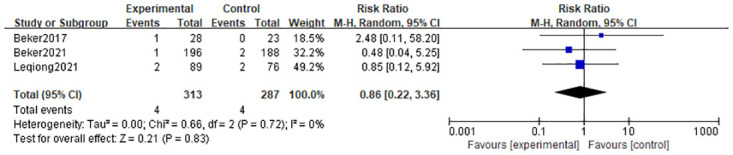
Forest plot for spontaneous intestinal perforation.

There was no difference in the relative risk of spontaneous intestinal perforation in the olfactory and/or gustatory stimulation group compared to standard care (RR = 0.86; 95% CI = 0.22, 3.36; P = 0.83) and there was no heterogeneity (I^2^ = 0%, P = 0.72) according to three studies [10, 22, 23] that examined the incidence of spontaneous intestinal perforation. (In Fig 9).

### 3.5 Publication bias and sensitivity analyses

We did not undertake funnel plot analysis to evaluate publication bias because there were only 7 studies. In addition, we conducted sensitivity analyses for each outcome by systematically excluding individual studies to help determine the stability and reliability of our conclusions. During the analysis, we found variations in the results for time to full enteral feeds and duration of parenteral nutrition. Thus, the stability and reliability of the results for complete enteral feeding time and parenteral nutrition duration were compromised.

### 3.6 Summary of the quality of the evidence

The overall strength of the evidence is rated as low to very low in this systematic review. There is a distinct "summary of findings" table for each comparison. (Table 2.) The overall quality of the evidence was rated as low for the incidence of necrotizing enterocolitis and the incidence of spontaneous bowel perforation for the comparison between the olfactory and/or gustatory stimulation intervention and standard care but as very low for time to full enteral feeds, the duration of parenteral feeding, the length of hospital stay, the weight at discharge, and the duration of gastric tube placement.

**Table 2:** Summary of findings: Olfactory and gustatory stimulation compared to standard care.

Certainty assessment							Summary of findings				
Participants (studies) Follow-up	Risk of bias	Inconsistency	Indirectness	Imprecision	Publication bias	Overall certainty of evidence	Study event rates (%)		Relative effect (95% CI)	Anticipated absolute effects	
							With exposure to the smell and taste	With Routine care		Risk with Routine care	Risk difference with exposure to the smell and taste
**Time to full enteral feeds**											
771 (6 RCTs)	serious	serious	serious	serious	publication bias strongly suspected all plausible residual confounding would reduce the demonstrated effect	⨁◯◯◯ Very low	375	396	-	-	MD **1.37 lower** (2.36 lower to 0.39 lower)
**Duration of parenteral nutrition**											
673 (4 RCTs)	serious	serious	serious	not serious	all plausible residual confounding would reduce the demonstrated effect	⨁◯◯◯Very low	325	348	-	-	MD **1.89 lower** (3.4 lower to 0.39 lower)
**Length of stay in hospital**											
391 (4 RCTs)	serious	very serious	serious	serious	all plausible residual confounding would reduce the demonstrated effect	⨁◯◯◯ Very low	189	202	-	-	MD **2.04 lower** (4.91 lower to 0.83 higher)
**Discharge weight**											
620 (5 RCTs)	serious	very serious	serious	serious	all plausible residual confounding would reduce the demonstrated effect	⨁◯◯◯ Very low	305	315	-	-	MD **19.63 lower** (183.87 lower to 144.61 higher)
**Duration of gastric tube placement**											
279 (2 RCTs)	serious	very serious	serious	serious	all plausible residual confounding would reduce the demonstrated effect	⨁◯◯◯ Very low	133	146	-	-	MD **1.85 lower** (3.99 lower to 0.28 higher)
**Necrotising enterocolitis**											
600 (3 RCTs)	serious	not serious	serious	serious	all plausible residual confounding would reduce the demonstrated effect	⨁⨁◯◯ Low	26/287 (9.1%)	24/313 (7.7%)	**RR 0.82** (0.48 to 1.39)	91 per 1,000	**16 fewer per 1,000** (from 47 fewer to 35 more)
**Spontaneous intestinal perforation**											
600 (3 RCTs)	serious	not serious	serious	serious	all plausible residual confounding would reduce the demonstrated effect	⨁⨁◯◯ Low	4/287 (1.4%)	4/313 (1.3%)	**RR 0.86** (0.22 to 3.36)	14 per 1,000	**2 fewer per 1,000** (from 11 fewer to 33 more)

## 4 Discussion

The purpose of this systematic review is to evaluate the impact of olfactory and/or gustatory stimulation interventions on feeding outcomes in preterm infants, including duration of parenteral nutrition, time to full enteral feeds, and secondary outcomes like length of hospital stay, discharge weight, duration of gastric tube retention, incidence of adverse events of necrotizing enterocolitis, and duration of gastric tube retention. Data from seven studies involving a total of 871 preterm infants are summarized in this study. The main findings suggest that olfactory and/or gustatory stimulation may reduce time to full enteral feeds in preterm infants when compared to standard care, and subgroup analysis revealed that either olfactory stimulation or both olfactory and gustatory stimulation may reduce the time to full enteral feeds in preterm infants. This suggests that odor and taste help facilitate digestion and nutrient metabolism in preterm infants. However, we still need to be skeptical about this result, as it showed instability in the sensitivity analysis. This could be explained by the significant variation in the studies’ findings, which could be attributed to variations in the preterm infants’ gestational ages. An earlier gestational age is typically associated with a more immature physical development, which raises the risk of extra-uterine growth restriction in preterm infants and, consequently, compromises the efficacy of the interventions in this study [27, 28]. Also, the heterogeneity may be related to the different frequencies of olfactory and gustatory stimuli during the intervention and the duration of the intervention. Some studies have shown that three-hour feeds are safe, as longer feeding intervals may improve blood flow and bowel motility after meals and help achieve complete enteral feeding earlier [29]. However, the studies included in this paper did not have a uniform duration and frequency of interventions, which leads to heterogeneity and thus affects the reliability of the results. This assessment investigated the potential effect of olfactory and gustatory stimuli on reducing the duration of parenteral nutrition in preterm infants, and the results suggest a potential role in reducing duration. However, the results of this study were shown to be unreliable in sensitivity analyses, which may be related to the small sample size and heterogeneity of the included studies, so the results need to be viewed with caution. Olfactory and gustatory stimulation did not affect the discharge weight of preterm infants when compared to usual care. Moreover, there is no indication that olfactory stimulation affects the duration of an indwelling gastric tube, the length of hospital stay, the incidence of necrotizing enterocolitis, or the incidence of spontaneous bowel perforation in premature children.

Olfactory and gustatory stimulation interventions represent a cost-effective and well-tolerated approach to expedite the initiation of sucking feeding in preterm infants. Extant research has demonstrated that neonates exhibit a predilection for amniotic fluid odors familiar to them within the initial three days postpartum [30]. This preference is intricately linked to the chemosensory exposures encountered by infants in the prenatal milieu [31]. Moreover, studies indicate that olfactory receptors retain enduring postnatal memories of the volatile constituents found in amniotic fluid. This retention enables infants to discern the distinct odors emanating from their mother’s breastmilk in contrast to those of other individuals [32]. Preterm infants, exposed to the scent of breast milk, demonstrated prolonged sucking sessions and increased feeding outbursts [31]. The substantiation of these findings is intricately linked to the observed phenomenon wherein olfactory and gustatory stimuli expedite suckling feeding in preterm infants. The results of this meta-analysis, however, deviate slightly from a recent study conducted by Muelbert [12]. The study by Muelbert demonstrated that exposure to olfactory and gustatory stimuli did not affect on time to full enteral feeds. Furthermore, it indicated a reduction in the length of hospital stay in preterm infants. We believe that the inconsistent results may be due to the limited number of research papers included in the previous meta-analysis, which consisted of only two trials involving 57 infants, and were therefore prone to significant bias. In contradistinction, the current study undertakes a more exhaustive examination of interventions involving olfactory and gustatory stimulation. This approach aims to facilitate the systematic comprehension of the effects of such interventions.

In preterm infants, mechanical functions such as sucking-swallowing coordination, gastric emptying, and intestinal motility are notably underdeveloped. This underdevelopment contributes to recurrent challenges, including issues with gastric residual aspirate, delayed meconium expulsion, and intestinal flatulence [33]. These difficulties frequently result in disruptions to established feeding schedules. Prolonged dependence on parenteral nutrition and insufficient enteral nutrition adversely affect preterm infants, potentially resulting in intestinal inflammation, atrophy, and sepsis during the neonatal period [34]. In addition, routine pre-feeding aspiration of gastric fluid to assess feeding tolerance and determine the amount to be fed is a common practice for feeding preterm infants [35]. However, evidence suggests that avoiding routine pre-feeding gastric aspirate monitoring can help reduce the incidence of late-onset sepsis, earlier achievement of complete enteral feeding, and earlier hospital discharge [36]. This is because frequent aspiration of gastric residue may cause gastric mucosal damage and intestinal bleeding [36]. As well as adversely affecting the protective microbiota, indirectly leading to the overgrowth of pathogenic bacteria [37]. Therefore, we need to focus on the effect of routine pre-feeding suctioning practices on the effectiveness of their interventions; however, the studies included in this paper did not detail whether the routine pre-feeding aspiration of gastric fluid was performed after olfactory and gustatory stimulation interventions. Therefore, the effects of each factor need to be considered together in future studies.

## 5 Limitations

Firstly, this study conducted a search of published literature in both Chinese and English languages. However, there might be incomplete article inclusion. Second, our study found moderate or high heterogeneity in the effect sizes of olfactory taste stimulation interventions. Despite our subgroup analyses based on intervention modality, the issue of heterogeneity remained unresolved. Sources of heterogeneity may result from varying definitions of outcome indicators, as well as different frequencies and durations of implementation of interventions. Finally, the sample sizes of the included studies were small and most of the studies had a high risk of bias.

## 6 Conclusions

Our review concluded olfactory and gustatory stimulation may reduce time to full enteral feeds and parenteral nutrition in preterm infants with no impact on the incidence of spontaneous intestinal perforation and necrotizing enterocolitis. Future studies should focus on utilizing standardized outcome measures and larger sample sizes to provide more reliable and conclusive evidence in this area.

## Supporting information

S1 File Example of a search formula. (DOCX)

S2 File Criteria for evaluating quality of evidence. (DOCX)

S3 File PRISMA-checklist. (DOCX)
