# Supplementary material for: Effects of olfactory and/or gustatory stimuli on feeding of preterm infants: A systematic review and meta-analysis
Source: PLoS One. 2024 May 7;19(5):e0301186. doi: 10.1371/journal.pone.0301186 (PMC11075836; doi:10.1371/journal.pone.0301186)

| **Pubmed** | | |
| --- | --- | --- |
| #1 | ((((Sense of Smell[Title/Abstract]) OR (Smell Sense[Title/Abstract])) OR (Olfaction[Title/Abstract]))) OR ("Smell"[Mesh]) | 24249 |
| #2 | (((Taste Sense[Title/Abstract]) OR (Gustation*[Title/Abstract])) OR (Senses, Taste[Title/Abstract])) OR ("Taste"[Mesh]) | 26572 |
| #3 | #1 and #2 | 48268 |
| #4 | (((Baby Formula*[Title/Abstract]) OR (Formula, Infant[Title/Abstract])) OR ("Infant Formula"[Mesh])) OR ((((Breast Milk[Title/Abstract]) OR (Human Milk[Title/Abstract])) OR ("Milk, Human"[Mesh])) OR ("Colostrum"[Mesh])) | 42935 |
| #5 | (((Premature Infant*[Title/Abstract]) OR (Preterm Infants[Title/Abstract])) OR (Neonatal Prematurity[Title/Abstract])) OR ("Infant, Premature"[Mesh]) | 82017 |
| #6 | #3 and #4 and #5 | 15 |

**Embase**


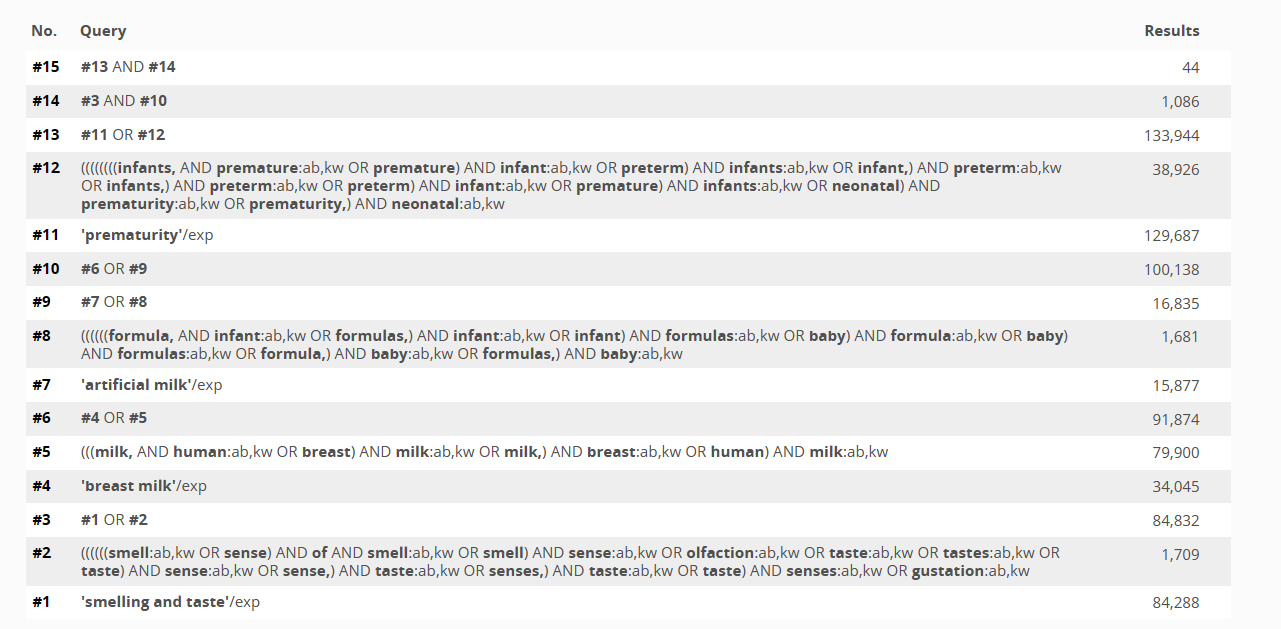

Supplement: S1 File — (DOCX) [file pone.0301186.s001.docx]
