# Supplementary material for: Effects of olfactory and/or gustatory stimuli on feeding of preterm infants: A systematic review and meta-analysis
Source: PLoS One. 2024 May 7;19(5):e0301186. doi: 10.1371/journal.pone.0301186 (PMC11075836; doi:10.1371/journal.pone.0301186)
Supplement: S2 File — (DOCX) [file pone.0301186.s002.docx]

| **Factors that reduce and improve the quality of evidence** | | | |
| --- | --- | --- | --- |
| **Study Design** | **Quality of Evidence** | **Lower if** | **Higher if** |
| Randomized trial | High | Risk of bias | Large effect |
|  |  | -1 Serious | ＋1 Large |
|  |  | -2 Very serious | ＋2 Very large |
|  | Moderate | Inconsistency | Dose response |
|  |  | -1 Serious | ＋1 Evidence of a gradient |
|  |  | -2 Very serious | All plausible confounding |
| Observational study | Low | Indirectness | ＋1 Would reduce a demonstrated effect |
|  |  | -1 Serious |  |
|  |  | -2 Very serious |  |
|  | Very low | Imprecision | ＋1 Would suggest a spurious effect when results show no effect |
|  |  | -1 Serious |  |
|  |  | -2 Very serious |  |
|  |  | Publication bias |  |
|  |  | -1 Likely |  |
|  |  | -2 Very likely |  |
